# Rising scabies incidence in Spain: a retrospective observational analysis of four national data sources, 2011 to 2023

**DOI:** 10.2807/1560-7917.ES.2025.30.47.2500296

**Published:** 2025-11-27

**Authors:** Allegra Chatterjee, Álvaro Roy, Cristina García-Blázquez, Israel Cruz, Diana Gómez-Barroso, Miguel Ángel Descalzo, Rosario Planelló, Zaida Herrador, SCABEAT Network group, SCABEAT Network, Ignacio Párraga Martínez, Remedios Martín Álvarez, Susana Aldecoa Landesa, Ignacio García Doval, Guadalupe Miró Corrales, Oscar Herrero Felipe, Mónica Aquilino Amez

**Affiliations:** 1 National Centre for Epidemiology, Instituto de Salud Carlos III (ISCIII), Madrid, Spain; 2 ECDC Fellowship Programme, Field Epidemiology path (EPIET), European Centre for Disease Prevention and Control (ECDC), Stockholm, Sweden; 3 Servicio de Medicina Preventiva, Hospital Universitario Clínico San Cecilio, Granada, Spain; 4 National School of Public Health, Instituto de Salud Carlos III (ISCIII), Madrid, Spain; 5 Networked Biomedical Research Centre in Infectious Diseases (CIBERINFEC), Madrid, Spain; 6 Networked Biomedical Research Centre in Epidemiology and Public Health (CIBERESP), Madrid, Spain; 7 Unidad de Investigación, Academia Española de Dermatología y Venereología (AEDV), Madrid, Spain; 8 Grupo de Entomología Molecular, Biomarcadores y Estrés Ambiental, Facultad de Ciencias, Universidad Nacional de Educación a Distancia (UNED), Las Rozas de Madrid, Spain; 9 The members of the network are listed under Collaborators.

**Keywords:** scabies, *Sarcoptes scabiei*, neglected tropical disease, Spain

## Abstract

**BACKGROUND:**

Scabies is a skin disease caused by the mite *Sarcoptes scabiei,* resulting in intense itching and rash, and sometimes secondary infections with complications. Scabies is not typically a notifiable disease, which makes estimating its burden of disease challenging. In recent years, sharp increases have been reported in Europe.

**AIM:**

This study characterises scabies epidemiology in Spain from 2011 to 2023.

**METHODS:**

This retrospective study triangulated data from primary care, hospital admissions, occupational diagnoses and outbreaks. Annual incidence rates (IRs) were calculated to assess temporal evolution, demographics and geographic distribution. Joinpoint regression identified IR changes, and time-series analysis explored seasonality. Occupational and outbreak data analysis identified high-risk activities and settings.

**RESULTS:**

Incidence rates increased across all data sources, with marked acceleration from 2020–21. The greatest rise was seen in primary care (annual percentage change rose from 22.8% (95% CI: 7.2–31.9) in 2011–20 to 65.8% (95% CI: 47.5–96.6) in 2020–23). The IR was highest amongst 15–24-year-olds. Hospitalisations, with highest IR among people > 65 years, peaked each January. Occupational diagnoses were predominantly registered in healthcare settings (82.0%). Islands and northern coastal regions were most affected. Outbreaks were most frequent in households and nursing homes, with largest outbreaks in healthcare settings.

**CONCLUSIONS:**

Given the increasing incidence of scabies in Spain, a strong response is needed to improve prevention, diagnosis, and treatment. Improved surveillance and targeted public health initiatives could mitigate further spread, as well as further research to better elucidate the mite-related and epidemiological factors that underline the recent increases across Europe.

**Key public health message**

**What did you want to address in this study and why?**

Scabies is a contagious yet neglected skin disease. Many countries in Europe have seen rises in cases and a shift from primarily affected vulnerable groups, such as institutionalised patients and people living in poor conditions, to the general population. Using four national data sources, we analysed scabies trends from 2011 to 2023 in Spain to identify groups and parts of the country that are most affected.

**What have we learnt from this study?**

Scabies has been rising across Spain since 2011, with a sharp acceleration from 2020. In primary care, cases rose on average by 66% annually from 2020–23, with a shift in the profile of affected individuals. Nursing assistants and other healthcare workers were the highest risk occupations and most outbreaks occurred in households or nursing homes.

**What are the implications of your findings for public health?**

Spain’s surge in scabies follows broader European trends and there is need for improved awareness among clinicians and the public to support prevention, early detection and treatment, particularly for risk groups, such as nursing homes residents and healthcare workers. Understanding potential drivers, including socioeconomic factors and treatment resistance, is important for public health action.

## Introduction

Scabies is a common contagious skin disease occurring globally [1] and caused by *Sarcoptes scabiei* (var. *hominis*), a parasitic mite that burrows into superficial skin layers and lays eggs. Symptoms include severe, persistent itching and rash, often resulting in sleep disturbance. Secondary bacterial infections (e.g. *Staphylococcus* and *Streptococcus*) of skin lesions are common, and can lead to severe complications including sepsis, soft-tissue necrosis, renal damage, rheumatic heart disease and, in some cases, death [2]. In classic scabies, patients are infected by an average of 10–15 mites with typically mild symptoms. However, some infections progress to a severe form of the disease, known as crusted scabies, where millions of mites may be present [3]. Crusted scabies and scabies-related complications are more common among immunocompromised individuals [2]. Transmission primarily occurs through prolonged skin-to-skin contact or, less frequently and generally limited to those with crusted scabies, through infested items such as bedding and clothes [2]. First-line treatment is typically topical permethrin, followed by or in conjunction with oral ivermectin.

The global prevalence of scabies is difficult to estimate, the World Health Organization (WHO) predicts that 200 million people worldwide have the disease at any given time [4]. Recognising the public health importance and historic lack of global attention, in 2017, scabies was added to the WHO list of Neglected Tropical Diseases (NTDs) [5]. In high-income countries, scabies has traditionally been associated with living in overcrowded and impoverished conditions, as well as institutional settings such as care homes and prisons [6]. In recent years, many countries including within Europe have seen large increases in scabies, with some experiencing exponential rises since the COVID-19 pandemic [7-11]. This has been accompanied by a shift in the profile of affected individuals, with the disease no longer confined to vulnerable groups or resource-poor settings, but increasingly affecting the general population [7].

As scabies is not a notifiable disease, obtaining a comprehensive epidemiological picture is challenging. Recent research suggests that Spain has been experiencing one of the largest scabies rises in Europe [10,12-15]. Data up to 2017 show an increase in scabies hospital admissions since 2014, following a decrease from the start of the study period in 1997 [10]. The present study provides updated and additional analyses using the same sources up to December 2023. In the context of rising scabies across Europe [10,12-16], we characterised scabies epidemiology in Spain in order to understand trends in incidence rates over time, demographic groups most at risk, spatial analysis with a time component and outbreak settings.

## Methods

### Study design

This retrospective observational study used data from four sources, each capturing information for distinct purposes and settings, extracted from 1 January 2011 to 31 December 2023: (i) primary care clinical records, (ii) hospital discharge records, (iii) occupational disease records and (iv) outbreak records. 

### Data sources and collection

The Primary Care Clinical Database (BDCAP in Spanish) collects anonymised data from a random sample of 12.9 million primary care patients (approx. 27% of the Spanish population) [17]. The BDCAP database was designed for statistical, research, clinical and administrative purposes, compiling codified and standardised information from primary care. BDCAP uses the International Classification of Primary Care (ICPC2), equivalent to the International Classification of Diseases (ICD) version 10 [18]. For this study, aggregated estimates of the number of appointments related to scabies were generated, weighted by autonomous community, sex and age group. We extracted primary care appointments coded with B86 (scabies), and medication prescribed through primary for years available (2017–23), using the Anatomical Therapeutic Chemical (ATC) code [19] P03A (ectoparasiticides including scabicides). Ivermectin was not included, as it falls under a different classification (P02CF01).

Databases for Hospital Discharge Records (CMBD and RAE-CMBD in Spanish) receive reports from ~98% of public and private hospitals. For 2011–16, we applied ICD-9 code 133.0 to CMBD, and for 2017–23, we applied ICD-10 code B86 for RAE-CMBD. We extracted hospitalisations where scabies was listed as either the primary (d1) or a secondary diagnosis (dx).

The Occupational Diseases Registry (CEPROSS in Spanish) records occupational diseases [20]. Occupation is classified according to the National Classification of Occupations (CNO11 in Spanish) [21], and exposure setting type according to the national codes for economic activities CNAE09 classification [22]. We applied ICD-10 code B86 to extract cases from CEPROSS.

The National Epidemiological Surveillance Network (RENAVE in Spanish) collects data on notifiable diseases from Spain’s 17 autonomous communities and two autonomous cities (Ceuta and Melilla). Scabies data reported to RENAVE is voluntary and limited to outbreaks with aggregated data. 

Additional details of the data sources are provided in Supplementary Table S1.

### Statistical analysis

We calculated frequencies and percentages to summarise data and used chi-square tests to assess whether sex and age distributions differed across data sources. For primary care and hospital data, we calculated mean annual incidence rates (IR) per million. Denominators used, obtained from the National Statistics Institute (INE) [23], were the total population (46.7–48.1 million) for primary care, hospital, and outbreak datasets, and the active population (23.4–24.1 million) for the occupational dataset. These denominators are provided in Supplementary Tables S4 and S5. The active population includes all people of working age who are either employed or unemployed but actively seeking work. To calculate IR by autonomous community we used primary care and hospital data for the full study period and for subperiods 2011–19 and 2020–23.

We characterised outbreaks for each setting by calculating the total number of outbreaks, number of cases per outbreak, secondary attack rate (AR) and duration. Aggregated sociodemographic information was available in BDCAP; scabies primary care appointments were analysed by rent level, economic activity, unemployment and country of birth.

Temporal trends of annual incidence rates were examined by fitting log-linear regression models using joinpoint software (https://surveillance.cancer.gov/joinpoint) to provide estimates of the annual percentage change (APC) in incidence rates, with corresponding 95% confidence intervals (CIs), and years where any trend changes have occurred.

We generated a predictive model based on monthly hospitalisation from 2011 to 2020 using a negative binomial regression that included terms for both trend and seasonality. To assess the presence of a temporal trend over this period, we first fitted a univariate negative binomial regression model with time (month and year) as the predictor and monthly aggregated hospitalisations as the outcome. Seasonality was incorporated by including sine and cosine terms derived from a Fourier series, allowing the model to capture recurring seasonal patterns. The model was fitted using the trending package in R, adapting code from The Epidemiologist R Handbook developed by Applied Epi [24]. Based on this predictive model, we estimated the expected number of monthly hospitalisations for 2021 and 2022, including 95% prediction intervals. 

We performed the analyses using Microsoft Excel and R (version 4.4.1). We created maps using QGIS software (version 3.36.0).

## Results

### Temporal assessment

Across the 13-year study period, 935,007 primary care appointments with a scabies diagnosis (mean annual IR = 1,517/million population), 6,068 hospital admissions with a scabies diagnosis (IR = 9.8/million), 3,942 occupational diagnoses (IR = 12.9/million) and 11,301 cases from 1,529 outbreaks (Table 1).

**Table 1:** Mean annual incidence rate of scabies by sex and age group for four data sources, Spain, 2011–2023

Characteristics		Primary care appointments (BDCAP) (n = 935,007)			Hospital admissions d1^a^ (CMBD) (n = 684)			Hospital admissions dx^a^ (CMBD) (n = 5,384)			Occupational diagnoses (CEPROSS^b^) (n = 3,942)			Outbreaks cases (RENAVE^c^) (n = 11,301)	
		n	%	IR	n	%	IR	n	%	IR	n	%	IR	n	%
Sex	Female	489,884	52.4	1,558.8	269	39.3	0.9	2,494	46.3	7.9	3,172	80.5	22.1	1,976	17.4
	Male	445,121	47.6	1,473.1	415	60.7	1.4	2,890	53.7	9.6	770	19.5	4.7	1,274	11.3
	Unknown	2	0.0	NA	0	0.0	NA	0	0.0	NA	0	0.0	NA	8,051	71.1
Age group in years^b,c^	0–14	191,561	20.5	2,190.0	185	27.1	2.1	729	13.5	8.3	0	0.0	NA	569	5.0
	15–24	209,599	22.4	3,310.8	47	6.9	0.8	529	9.8	8.4	351	8.9	16.7	339	3.0
	25–44	223,581	23.9	1,379.7	88	12.9	0.5	769	14.3	4.7	1,816	46.1	12.7	638	5.6
	45–64	212,620	22.7	1,167.8	143	20.9	0.8	1,282	23.8	7.1	1,752	44.5	12.8	480	4.2
	≥ 65	97,646	10.4	805.5	220	32.2	1.9	2,075	38.5	17.2	21	0.5	5.5	1,403	12.4
	Unknown	0	0.0	NA	1	0.0	NA	0	0.0	NA	2	0.0	NA	7,872	69.7

BDCAP: Primary Care Clinical Database; CEPROSS: Occupational Diseases Registry; CI: confidence interval; CMBD: Hospital Discharge Records; IR: mean annual incidence rate per million population; NA: not applicable; RENAVE: Spanish National Epidemiological Surveillance Network.

ᵃ d1 refers to hospitalisations where scabies was the primary diagnosis at admission; dx refers to hospitalisations where scabies was a secondary diagnosis at admission.

^b^ As CEPROSS reports cases amongst the working population, there are no children included so the first age category is 16–24 years (age 15 not included).

^c^ RENAVE incidence rates were not included because of the large amount of missing demographic data.

Data sources are as follows: primary care appointments (BDCAP), hospital admissions (CMBDᵃ), occupational diagnoses (CEPROSS^b^), and outbreak cases (RENAVE^c^).

**Figure 1:**
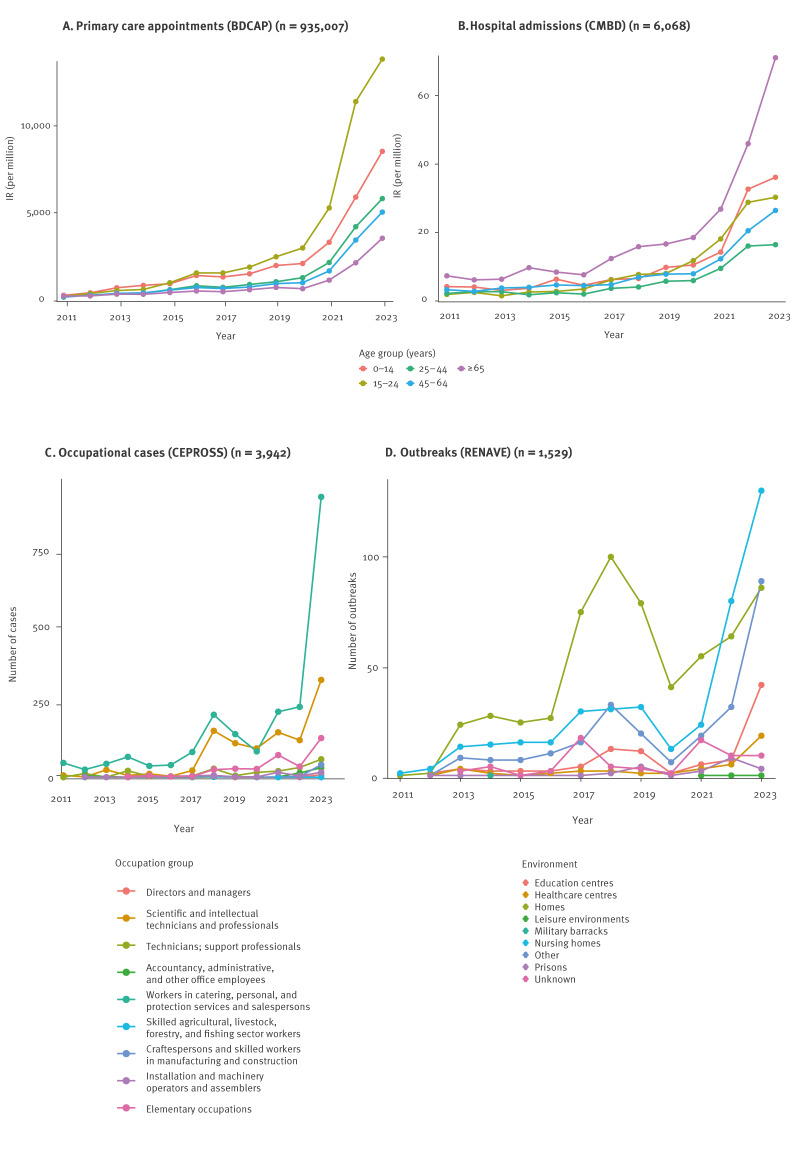
Annual scabies incidence rate of (A) primary care appointments and (B) hospital admissions by age group, (C) number of cases reported by occupational group and (D) number of outbreaks by setting type, Spain, 2011–2023

BDCAP: Primary Care Clinical Database; CEPROSS: Occupational Diseases Registry; CMBD: Hospital Discharge Records; RENAVE: Spanish National Epidemiological Surveillance Network.

A rising trend in incidence was observed across all datasets (Figure 1). The mean annual percentage change (APC) ranged from 14% (95% CI: 10–21) for hospital admissions with scabies as a primary diagnosis to 32% (95% CI: 28–39) for primary care appointments (Table 2). Comparing the beginning and end of the study period, the primary care annual IR increased from 130.9 per million in 2011 to 6,307.3 in 2023; representing nearly a 50-fold rise.

Incidence rates calculated per million population for primary care, hospitalisations and outbreaks, and per million active population (16 years and above) for people with an occupational diagnosis. The active population includes all people of working age who are either employed or unemployed but actively seeking work.

**Table 2:** Estimates of annual percentage change in scabies incidence rates determined by joinpoint regression, by data source, Spain, overall (2011–2023) and subperiods (2011–2020 and 2020–2023)

Data source	n	Period	APC	95% CIs	p values
Primary care appointments (BDCAP)	n = 935,005	Overall	32.4	27.9 to 38.7	<0.001
		2011–20	22.8	7.2 to 31.9	0.021
		2020–23	65.8	47.5 to 96.6	<0.001
Hospital admissions, d1 and dx combined^a^ (CMBD)	n = 6,068	Overall	25.7	23.1 to 29.2	<0.001
		2011–20	17.4	10.6 to 22.5	0.002
		2020–23	54.3	41.2 to 76.1	<0.001
Hospital admissions, d1^a^ (CMBD)	n = 684	Overall	13.9	10.2 to 21.1	<0.001
		2011–14	−14.5	−42.6 to 14.5	0.406
		2014–23	25.3	20.7 to 44.2	<0.001
Hospital admissions, dx^a^ (CMBD)	n = 5,384	Overall	26.7	24.3 to 30.2	<0.001
		2011–20	18.2	12.3 to 23.1	<0.001
		2020–23	56.3	44.2 to 77.0	<0.001
Occupational diagnoses (CEPROSS)	n = 3,942	Overall	31.3	12.3 to 53.5	<0.001
		2011–21	23.3	5.8 to 43.8	0.014
		2021–23	79.3	−18.8 to 295.5	0.127
Outbreaks (RENAVE)	n = 11,301 cases, 1,529 outbreaks	Overall	17.3	0.3 to 37.1	0.046
		2011–21	11.4	−4.6 to 30.0	0.150
		2021–23	51.8	−31.1 to 234.1	0.260

APC: mean annual percentage change; BDCAP: Primary Care Clinical Database; CEPROSS: Occupational Diseases Registry; CI: confidence interval; CMBD: Hospital Discharge Records; RENAVE: Spanish National Epidemiological Surveillance Network.

^a^ d1 refers to hospitalisations where scabies was the primary diagnosis at admission; dx refers to hospitalisations where scabies was a secondary diagnosis at admission.

Joinpoint linear regression was performed using the National Cancer Institute software. This technique provides estimates of the annual percentage change (APC) in incidence rates, with corresponding 95% confidence intervals (CIs), and tests whether any apparent changes in trend are statistically significant using a Monte Carlo Permutation method [25]. Years where joinpoint trend changes were detected vary for each data source. Additional details about IR per million population for each data source for the whole period (2011–23) and subperiods are provided in Supplementary Table S2.

Joinpoint regression indicated that the rate of increase accelerated from 2020−21 for all sources; in primary care the APC for years 2011–20 was 23% (95% CI: 7–32), rising to 66% (95% CI: 48–97) for years 2020–23 (Table 2). Hospital admissions (primary and secondary diagnoses combined) mirrored these trends, with APCs of 17% (95% CI: 11–23) and 54% (95% CI: 41–76), respectively. Occupational diagnoses and outbreaks also showed sharper rises from 2021 (Figure 1C and 1D); however, these changes were not statistically significant (Table 2).

By age, the 15–24 years group had the highest primary care IR and greatest rise in IR (Figure 1A), with a mean APC of 40% (95% CI: 30–48); however, confidence intervals overlapped somewhat with other age groups. Among hospital admissions, the largest increase was observed in those aged over 65 years (Figure 1B), who also had the highest IR overall across the study period (Table 1).

Changes in the profile of occupational and outbreak cases were also seen. The greatest proportion of cases and largest rise was observed among workers in catering, personal, and protection services and salespersons [21] (Figure 1C), comprising 55% of occupational diagnoses (2,162/3,942) and rising 11-fold between years 2020 and 2023 from 86 to 926 cases. This change was mostly driven by cases amongst nursing assistants who comprised 59% (1,282/2,162) of this group and 32% (1,281/3,942) of all occupational diagnoses. These nursing assistants were predominantly hospital-based (47%; 1,019/2,162) with the remainder in primary care (12%; 262/2,162). The second most affected occupation group was scientific and intellectual technicians and professionals, which includes nurses, doctors, and other professional health workers along with some other professions, which comprised 26% (1,037/3,942) of cases, and has also seen a large increase since 2020 (Figure 1C).

The setting where the greatest rise in outbreaks has occurred is nursing homes, with a 10-fold increase from 13 to 130 between 2020 and 2023. A peak in household outbreaks was seen in 2018 (100 outbreaks), before declining until 2020 (n = 41) and rising again until 2023 (n = 86), but still below 2018 levels (Figure 1D).

The number of people prescribed an ectoparasiticide per thousand population and the daily defined dose (DDD) per 1,000 people who attended primary care per day from 2017–23 in Spain is provided in Supplementary Table S3. A substantial increase in ectoparasiticide prescriptions within primary care was observed. The DDD per thousand people attending primary care per day increased from 0.4 in 2017 to 3.8 in 2023 (10-fold rise), with a mean APC of 47%. The number of people receiving treatment also increased from 20,763 in 2017 to 324,287 in 2023 (16-fold increase), with a mean APC of 58%.

**Figure 2:**
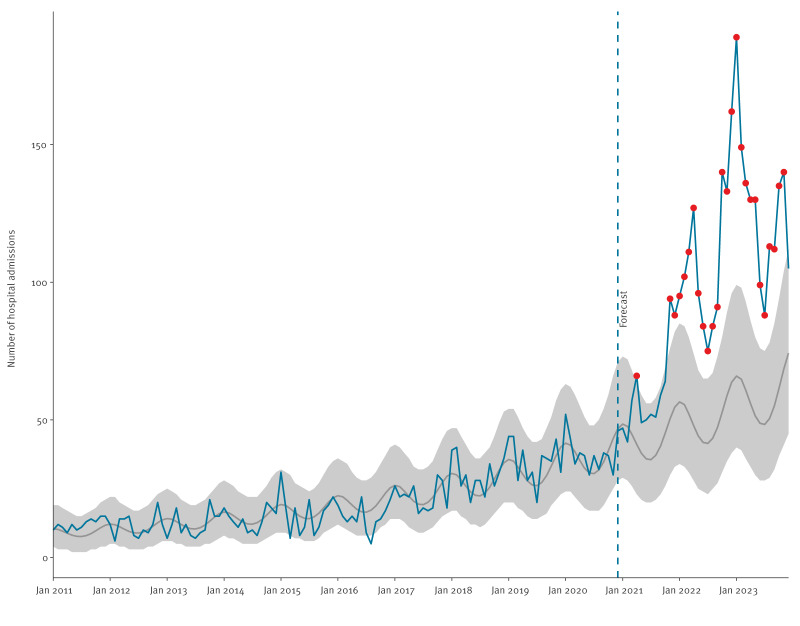
Monthly number of hospital admissions with scabies as a primary or secondary cause compared to the forecasted monthly hospitalisations based on 2011–2020 notifications, Spain, 2011–2023 (n = 6,068)

Seasonal patterns in hospital admissions were observed, with peaks occurring every January, except years 2012 and 2013 where hospitalisations appeared to peak in the months prior to January when they were lowest (Figure 2). In 3 months of 2021 and all months of 2022 and 2023, the number of admissions exceeded the 95% prediction interval of the model.

Blue line: reported hospital admissions; grey line: predicted hospital admissions; shaded area: 95% prediction interval; red dots: months when hospital admissions were above the 95% prediction interval.

### Sociodemographic and clinical characteristics

Chi-square tests indicated significant differences in sex distribution between the four data sources (p < 0.001). There was a slightly higher proportion of women (52%) than men in primary care, and a strong female predominance amongst people with occupational diagnoses (81%) (Table 1). In contrast, 54% of hospital admissions were men, with an even stronger male predominance where scabies was primary diagnosis (61%).

Age group distribution also differed by data source (p < 0.001). Among primary care contacts, the average annual IR was highest for age 15–24 years, and lowest in the oldest group of those aged over 65 years (3,311 vs 806 per million, respectively) (Table 1). Hospital admissions with a primary scabies diagnosis had the highest IR among the youngest age group, 0–14 years (IR = 2.1), followed by the oldest (≥ 65 years, IR = 1.9). Amongst admissions with a secondary scabies diagnosis, IR was highest among the oldest age group (IR = 17.2). Similarly to primary care, IR among people with occupational diagnoses was highest amongst those aged 16–24 years (IR = 16.7).

Over the study period, 80% (750,782/935,005) of primary care appointments were among individuals with rent levels of below EUR 18,000 per year and 31% (288,998/935,005) were ‘economically active’. However, there appear to be changing trends; the proportion of appointments among people who are economically active has risen from a low of 24% (4,133/17,046) in 2013 to 34% (103,123/303,290) in 2023, with the greatest change seen since 2020 (28%, 17,047/61,542). In line with this, the proportion of primary care appointments among people without unemployment has almost halved from 2020 (12%, 7,252/61,542) to 2023 (6.5%, 103,123/303,290). Among individuals with recorded birth origin (91% of primary care appointments), 18% (154,996/854,437) were born outside of Spain, and there does not appear to be any time trend.

### Geographic distribution

**Figure 3:**
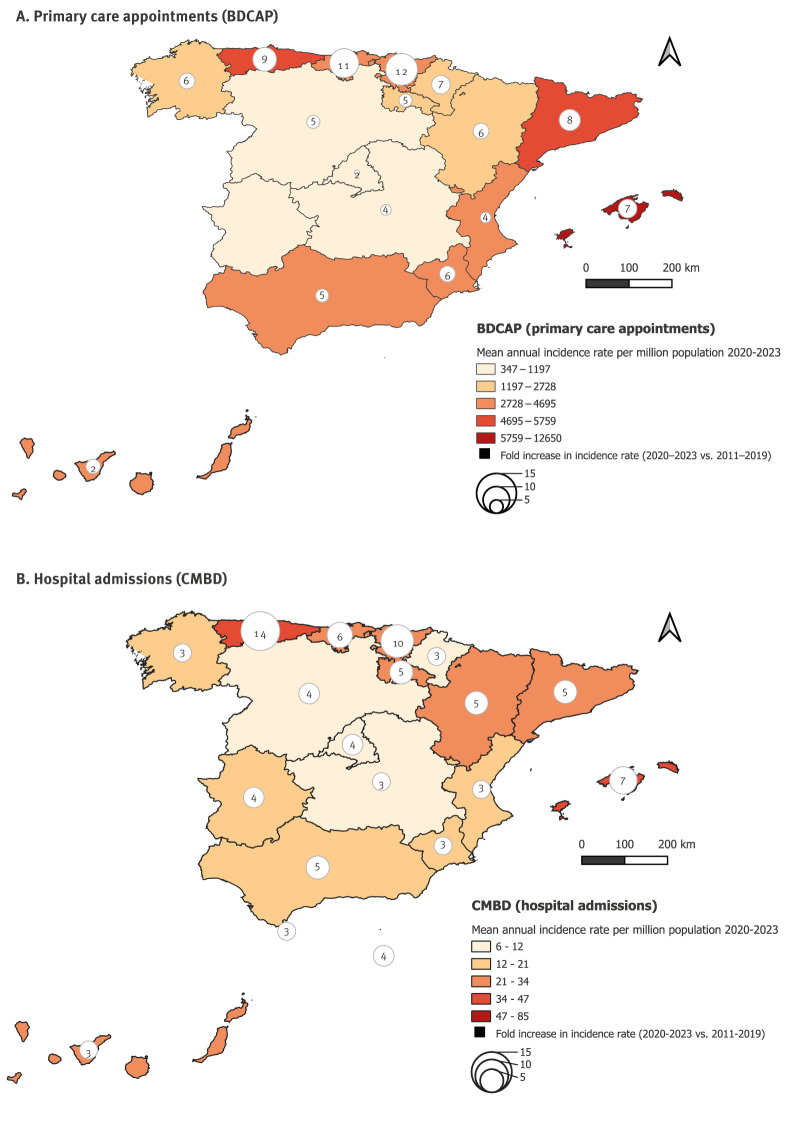
Mean annual scabies diagnoses incidence rate per million of (A) primary care appointments and (B) hospital admissions by autonomous community between 2020–2023 and percentage change compared with period 2011–2019, Spain

BDCAP: Primary Care Clinical Database; CMBD: Hospital Discharge Records.

Across the study period, the highest mean annual primary care IR occurred in island and coastal regions, particularly the Balearic Islands (5,209/million) and Canary Islands (2,971/million) followed by north-west regions such as Asturias (2,489/million) (Figure 3). Similarly, the Balearic Islands and Asturias reported some of the highest hospital admissions (both with IR = 17/million), lower only than Melilla (IR = 52/million) and Ceuta (IR = 44/million) (data not shown), for which primary care data were not available.

The colours represent the mean annual scabies appointments (BDCAP) and hospital admissions (CMBD) incidence rate per million population during 2020–23. The circles represent the fold increase in incidence when comparing the 2020–23 period with the 2011–2019 period. The number inside each circle indicates the fold increase, and the circle size is proportional to its value.

Inland autonomous communities reported the lowest primary care IRs, including Extremadura (IR = 126/million) and Castilla y León (IR = 536/million). Similarly, autonomous communities in the centre of the country reported the lowest hospitalisations; Castilla-La Mancha (IR = 3.1/million), Castilla y León (IR = 3.4/million), and Madrid (IR = 5.1/million).

All autonomous communities saw large increases in annual IR during 2020–23 relative to 2011–19, with a national average six-fold increase in incidence in primary care contacts and a five-fold increase in hospital admissions. The greatest primary care increases were in the Basque Country (12-fold), Cantabria (11-fold), and Asturias (9-fold), with the smallest in the Canary Islands and Madrid (2-fold). Similarly, Asturias and the Basque Country also saw the greatest increases in hospital admission IRs (14-fold and 10-fold, respectively), with the smallest rises in Galicia and Valencia (3-fold) (Figure 3).

### Outbreaks

Households were the most common outbreak setting, accounting for 40% (n = 607) of reported outbreaks, comprising 23% (n = 2,604) of outbreak-related cases, with a mean of four individuals with scabies diagnoses per outbreak. Following this, nursing homes represented 27% (n = 407) of outbreaks, contributing the highest total number of cases (46%, n = 5,226), with 13 cases per outbreak. Although there were only two outbreaks in military barracks, this setting reported the highest mean number of cases per outbreak (n = 57), followed by nursing homes (n = 13) (Table 3).

**Table 3:** Scabies outbreaks reported to the National Epidemiological Surveillance Network (RENAVE), Spain, 2011–2023 (n = 1,529)

Setting	Outbreaks		Individuals		Mean cases per outbreak	Median attack rate^a^			Median duration^a^		
	n	%	n	%		n	%	IQR	n	days	IQR
Households	607	39.7	2,604	23.0	4.3	299	75.0	57.1–100	301	31.0	7.0–88.0
Nursing homes	407	26.6	5,226	46.2	12.8	294	11.3	5.0–23.2	255	29.0	8.5–77.5
Education centres	103	6.7	490	4.3	5.3	53	14.3	7.7–33.3	57	20.0	5.0–62.0
Healthcare settings	49	3.2	442	3.9	9.0	20	22.6	7.7–100	19	54.0	0.5–73.5
Prisons	27	1.8	204	1.8	7.6	11	50.0	18.2–100	16	15.0	3.2–62.5
Leisure settings	3	0.2	16	0.1	5.3	3	25.0	20.2–37.5	1	10.0	10.0–10.0
Military barracks	2	0.1	114	1.0	57.0	2	51.4	27.1–75.7	2	30.5	27.2–33.8
Other	254	16.6	1,657	14.7	6.5	180	16.7	7.3–35.7	135	26.0	7.0–60.5
Unknown	77	5.0	548	4.8	7.1	23	29.2	8.6–71.4	35	43.0	25.5–84.0
**Total**	**1,529**	**100**	**11,301**	**100**	**7.4**	**885**	**29.3**	**8.6**–**71**	**821**	**29.0**	**7.0**–**77.0**

IQR: interquartile range.

^a^ The number of outbreaks with data available varies for each setting and are listed in brackets. For all settings combined, data are available to calculate secondary attack rates for 57.9% outbreaks, and duration for 53.7% outbreaks.

The median secondary AR was 29%. However, information was only available for 58% (885/1,529 of reported outbreaks). The highest AR was observed in households (75%), followed by military barracks (51%), with the lowest AR occurring in nursing homes (11.3%). Median duration across all settings was 29 days (IQR: 7–77), with longest outbreaks in healthcare settings 54 days (IQR: 0.5–74). Excluding leisure settings where duration was reported for only one outbreak, the shortest duration was in prisons (median: 15 days; IQR: 3–63) (Table 3).

## Discussion

A rising trend in scabies incidence was observed across all data sources during the 13-year period from 2011 to 2023 in Spain. This builds on findings from previous studies in Spain, including one using the same four sources with data up to 2019 [10], and another which reported a six-fold increase in permethrin prescriptions from 2008 to 2021 [15]. This also aligns with broader patterns across Europe; a 2022 review including studies from 15 European countries concluded that scabies has been rising in all contexts [7]. Although differences in surveillance and reporting makes direct comparison difficult, overall trends were consistent; for example, a ninefold increase in incidence in Germany between 2009–18 [26], threefold in the Netherlands between 2011–20 [9], and sixfold in Croatia between 2007–17 [27].

Our study found a rise in incidence since 2020. Some previous studies reported a decline in scabies during the COVID-19 pandemic [28], possibly reflecting the true burden or underreporting because of reduced non-emergency care access. Others, however, including small studies in a Spanish hospital [12] and United Kingdom (UK) primary care practice [29], found increased incidence during lockdowns, possibly driven by greater household transmission. In our data, although cases continued to rise through the pandemic, the number of household outbreaks dropped in 2019–20, possibly due to underreporting. While there is currently limited research from other European countries covering years following the pandemic, studies that have been published show similar trends to Spain, for example, in Sicily [30] and Lazio [28], Italy, where cases have surged since 2020.

Multiple factors may contribute to this increase. A surge in tourism and use of shared accommodation, particularly post-pandemic, have been suggested in other studies such as one from Bologna [31]. Spain, the world’s second most visited country, saw a 5-fold increase in tourism income from 2020 to 2023 [32], which may have contributed to transmission. Treatment resistance may also play a role, with evidence from many contexts suggesting declining permethrin efficacy, and possible ivermectin resistance particularly for crusted scabies [33]. 

Another explanation might be the lack of a standardised diagnostic test for scabies and that symptoms, which in addition can overlap with other dermatological conditions. Although to our knowledge no studies have assessed the diagnostic accuracy of scabies in Spain, dermoscopy is commonly used which has high sensitivity (98%) and specificity (89%) [34]. 

January peaks in scabies-related hospital admissions align with previous research linking higher incidence to lower temperatures and increased humidity, conditions which promote indoor crowding and may enhance mite survival and fertility [35]. Similarly, higher permethrin prescription rates have been observed during winter months in Spain [15]. However, further analysis is needed to determine whether observed peaks exceed expected seasonal trends in hospitalisations after accounting for other causes.

The highest IR and fastest rate of increase in primary care was observed among adolescents and young adults (15–24 years), mirroring trends in other European countries including Germany [26] and the Netherlands [9]. Unemployment levels among primary care appointments also appear to have reduced over time. This represents a substantial shift in the profile of scabies cases, where in high-income countries the disease has primarily affected those living in poverty or institutional settings. The reasons underlying these demographic shifts remain unclear, however, changes in household structure may contribute. Across Europe, the substantial decline in home ownership among under 35-year-olds in recent decades [36] means that in Spain a large and increasing proportion of young adults live in shared rented accommodation for longer periods [37]. Our data show that households have the highest scabies attack rate (75%) and are the setting where most outbreaks occur, therefore changes in household structure may contribute to the changes in scabies case demographics. Sexual transmission may also be a factor as it is a known transmission route, particularly among young people [38]. Indeed, across the study period, trends in other sexually transmitted infections (STI), such as gonorrhoea and syphilis [39] in Spain have followed a similar pattern to scabies. 

While there is no ICD10 code specifically for crusted scabies, hospital admissions with scabies as a primary diagnosis (d1) may serve as a proxy for severe cases since milder forms rarely require hospitalisation. The highest incidence in our data was observed amongst children (0–14 years), aligning with international evidence that children often experience more severe disease and complications [7]. Admissions with scabies as a secondary diagnosis were highest among those aged ≥ 65 years, which may reflect delayed diagnoses that are only made when patients seek care for other conditions. However, further analysis is needed to understand to what extent this simply reflects that elderly people over 64 years are more likely to be hospitalised, leading to higher rates of secondary scabies diagnosis.

Our analysis indicated that occupational diagnoses were predominantly in health and social care workers**,** a sector that has also seen the largest increase in recent years. Nursing assistants accounted for over one-third of cases**,** reflecting the high exposure through direct patient contact. The overwhelming female predominance (81%) among people with an occupational diagnosis likely reflects the disproportionate representation of women in care roles. In line with this, nursing homes had the second highest outbreak numbers after households**,** consistent with findings from other European countries [28].

Regional differences in scabies incidence within Spain were striking. The reasons remain unclear and may involve climate, sociodemographic drivers, healthcare access, or other factors. While scabies is historically associated with living in overcrowded and poor conditions, its Spanish distribution does not align with this. The Balearic Islands, Canary Islands, and Asturias had the highest primary care burden, yet ranked eighth, third, and twelfth**,** respectively, in poverty levels among the 17 autonomous communities [40].

The largest post-COVID-19 increases occurred in the Basque Country, Cantabria, and Asturias**,** adjoining northern coastal regions. Further research is needed to explore the underlying causes, for example whether it may be related to rises in tourism in these areas or the impact of climate factors as mites prefer cooler and more humid conditions. 

Our study had some limitations. This analysis relied on four data sources designed for different purposes with their own limitations. BDCAP provides valuable primary care insights but lacks individual-level sociodemographic data. While it captures permethrin prescriptions, ivermectin, which has only been approved by the Spanish drug agency since 2021 [41], is not included, limiting treatment analysis. CMBD hospital admissions serve as a proxy for severe cases, including crusted scabies, given the lack of subclassification coding. CEPROSS identifies high-risk professions, supporting occupational health interventions. RENAVE provides insights into outbreak characteristics including duration and attack rates but varies in reporting completeness, limiting demographic analyses. A key challenge is the lack of unique patient identifiers, preventing cross-linking across sources. Nevertheless, inclusion of data from 2011–23 across all sources ensures robust temporal comparability and provides key insights into post-pandemic trends, which remain underexplored.

## Conclusion

Given the increasing incidence of scabies across Spain, a strong response is needed to improve prevention, diagnosis and treatment. Engaging the public through community-led initiatives focussing on those most at risk, including adolescents and young adults and the elderly population, could enhance awareness and early detection. In particular, raising awareness among young people of sexual contact as a possible transmission route for scabies. Clinician training should focus on improved diagnosis and use of personal protective equipment to reduce nosocomial transmission. Enhancing surveillance would improve epidemiological monitoring and outbreak detection. Finally, continued collaboration among public health authorities, clinicians, researchers, and the wider community is essential to reducing the scabies burden and improving public health.

## Data Availability

This study involves the use of patient data owned by third-party organisations. The BDCAP and CMBD is a set of clinical-administrative data of hospitalisations that collects sociodemographic, clinical and administrative patient data from the National Health System (NHS) discharge reports. It is hosted by the Ministry of Health (https://www.sanidad.gob.es/estadEstudios/portada/home.htm). The National Epidemiological Surveillance Network (RENAVE) provides data on cases reported through the national reporting electronic platform (in Spanish Sistema de Vigilancia en España - SiViEs) and it is hosted by the National Centre of Epidemiology (https://cne.isciii.es/es/servicios/enfermedades-transmisibles/protocolos-renave). CEPROSS data are available upon request in the following link: https://sede.seg-social.gob.es/wps/portal/sede/sede/AdministracionesyMutuas/13CEPROSS_1/21684EP. All sources follow the mandate of Spanish and international legislation.

## SCABEAT Network collaborator group

SCABEAT Network: Scabies Control and Action through Behavior, Epidemiology, Awareness, and Treatment. Sociedad Española de Medicina de Familia y Comunitaria (semFYC), Spain: Ignacio Párraga Martínez, Remedios Martín Álvarez, Susana Aldecoa Landesa. Unidad de Investigación, Academia Española de Dermatología y Venereología (AEDV), Spain: Ignacio García Doval. Facultad de Veterinaria, Universidad Complutense de Madrid, Madrid, Spain: Guadalupe Miró Corrales. Grupo de Entomología Molecular, Biomarcadores y Estrés Ambiental, Facultad de Ciencias, Universidad Nacional de Educación a Distancia (UNED), Madrid, Spain: Oscar Herrero Felipe, Mónica Aquilino Amez.

## Supplementary Data

288000 Supplement
