# Supplementary material for: Rising scabies incidence in Spain: a retrospective observational analysis of four national data sources, 2011 to 2023
Source: Euro Surveill. 2025 Nov 27;30(47):2500296. doi: 10.2807/1560-7917.ES.2025.30.47.2500296 (PMC12645275; doi:10.2807/1560-7917.ES.2025.30.47.2500296)
Supplement: Supplement [file 25-00296_HERRADOR_Supplement.pdf]

This supplementary material is hosted by *Eurosurveillance* as supporting information alongside the article "Rising scabies incidence in Spain, 2011–2023: a retrospective observational analysis of four national data sources", on behalf of the authors, who remain responsible for the accuracy and appropriateness of the content. The same standards for ethics, copyright, attributions and permissions as for the article apply. Supplements are not edited by *Eurosurveillance* and the journal is not responsible for the maintenance of any links or email addresses provided therein

Supplementary Table S1. Data sources used (BDCAP, CMDB, CEPROSS, RENAVE) and characteristics.

Database	Records	Coverage	Information
Primary Care Clinical Database (BDCAP in Spanish)	Primary care contacts	Sentinel sites, nationally representative (~27% sample)	Demographics, rent level, employment status, prescribed medication
Hospital Discharge Records (RAE-CMBD in Spanish) and the Minimum Basic Data Set of Hospital Discharges (CMBD in Spanish)	Hospital admissions	National	Demographics, diagnosis on admission (primary and secondary reason)
Occupational Diseases Registry (CEPROSS in Spanish)	Occupational diseases recorded in the National Social Security System	National	Demographics, occupation, work setting
The National Surveillance Network (RENAVE in Spanish)	Outbreaks recorded in the National Surveillance Network	National (not mandatory)	Demographics, primary case number of contacts, attack rate, outbreak duration,

			outbreak environment

Supplementary Table S2. Mean annual rate (IR) per million population for each data source for the whole period (2011-2023) and subperiods in Spain.

Source	Period	Mean annual IR /million
Primary care (BDCAP)<br>(N=935,007)	Overall (2011-23)<br>2011-19<br>2020-23	1517<br>598<br>3585
Hospital, d1 and dx combined [a]<br>(CMBD)<br>(N=6,068)	Overall (2011-23)<br>2011-19<br>2020-23	9.9<br>4.7<br>21
Hospital, d1 [a] (CMBD)<br>(N=684)	Overall (2011-23)<br>2011-13<br>2014-23	1.1<br>0.5<br>1.3
Hospital, dx [a] (CMBD)<br>(N=5,384)	Overall (2011-23)<br>2011-19<br>2020-23	8.7<br>4.0<br>19
Occupational (CEPROSS)<br>(N=3,942)	Overall (2011-23)<br>2011-20<br>2021-23	13<br>6.4<br>35
Outbreaks (RENAVE)<br>(N=11,301 cases, 1,529 outbreaks	Overall (2011-23)<br>2011-20<br>2021-23	18<br>13<br>38

[a] d1 = hospitalisations where scabies was the primary diagnosis at admission; dx = hospitalisations where scabies was the secondary diagnosis at admission.

Supplementary Table S3. The number of people prescribed an ectoparasiticide [a] per thousand population and the daily defined dose (DDD) per thousand people who attended primary care per day from 2017-2023 in Spain.

Year	Number of people prescribed ectoparasiticide [a]	Number of people prescribed ectoparasiticide [a] /thousand	DDD/thousand attended per day
2017	20,763	0.89	0.37
2018	46,340	1.4	0.66
2019	81,322	1.8	0.82
2020	87,222	1.92	1
2021	137,543	3.00	1.73
2022	251,259	5.43	2.9
2023	324,287	6.93	3.76

[a] Anatomical Therapeutic Chemical (ATC) code P03A

Sex	Year	Age (years)					Total
		0-14	15-24	25-44	45-64	>=65	
Male	2011	3,625,253	2,428,328	7,673,646	5,906,798	3,439,266	23,073,291
	2012	3,648,088	2,380,352	7,500,646	6,025,061	3,501,602	23,055,749
	2013	3,642,381	2,330,791	7,267,672	6,111,538	3,581,380	22,933,762
	2014	3,632,107	2,297,600	7,052,823	6,197,184	3,660,371	22,840,085
	2015	3,621,324	2,287,320	6,869,172	6,303,601	3,719,458	22,800,875
	2016	3,609,698	2,291,467	6,704,200	6,420,949	3,778,946	22,805,260
	2017	3,596,959	2,305,323	6,552,991	6,533,041	3,841,438	22,829,752
	2018	3,578,309	2,341,567	6,436,852	6,646,873	3,908,086	22,911,687
	2019	3,554,014	2,406,990	6,359,288	6,786,763	3,984,848	23,091,903

	2020	3,515,168	2,467,903	6,261,768	6,920,156	4,043,812	23,208,807
	2021	3,436,964	2,505,972	6,105,228	7,030,553	4,110,261	23,188,978
	2022	3,415,603	2,537,700	6,045,499	7,088,879	4,149,350	23,237,031
	2023	3,364,448	2,610,541	6,132,869	7,244,195	4,213,540	23,565,593
Female	2011	3,414,765	2,318,714	7,312,627	6,003,950	4,612,933	23,662,989
	2012	3,433,907	2,275,764	7,201,853	6,120,881	4,678,281	23,710,686
	2013	3,428,222	2,226,346	7,032,872	6,207,874	4,764,161	23,659,475
	2014	3,417,649	2,191,366	6,864,861	6,291,037	4,850,104	23,615,017
	2015	3,407,000	2,177,801	6,717,933	6,395,203	4,911,340	23,609,277
	2016	3,395,477	2,178,146	6,584,630	6,512,666	4,973,690	23,644,609
	2017	3,383,012	2,190,274	6,463,294	6,629,060	5,037,507	23,703,147
	2018	3,364,635	2,222,010	6,373,323	6,749,655	5,107,578	23,817,201
	2019	3,341,443	2,275,224	6,309,382	6,895,059	5,192,361	24,013,469
	2020	3,305,425	2,326,849	6,223,180	7,033,919	5,257,563	24,146,936
	2021	3,232,049	2,353,657	6,074,563	7,143,822	5,333,784	24,137,875
	2022	3,211,628	2,379,785	6,020,170	7,206,299	5,377,933	24,195,815
	2023	3,172,767	2,455,255	6,065,001	7,352,509	5,474,236	24,519,768

Supplementary Table S4. Active population used for occupational case calculations, Spain, 2011-2023. Data extracted from the Spanish Statistics Office (Instituto Nacional de Estadística, INE).

**Year**	**Population**
2011	23,434,075
2012	23,443,600
2013	23,190,075
2014	22,954,650
2015	22,922,000
2016	22,822,700
2017	22,741,800
2018	22,806,700
2019	23,027,050
2020	22,733,225
2021	23,309,875
2022	23,626,625
2023	24,119,725
